# Changes after Death

**Published:** 1889-10-19

**Authors:** 


### JURISPRUDENCE.

### CHANGES AFTER DEATH.

Dr. Coull Mackenzie, the police surgeon, Calcutta, publishes in a recent number of the *Indian Medical Gazette* some notes on "Phenomena Occurring After Death." The observations are interesting, as showing the differences in such matters in Europe and in a tropical country. For instance, in Europe the period of irritability or contractility of dead bodies lasts from a few minutes to twenty-four hours after death. In Calcutta the longest period was four hours and thirty minutes. Cadaveric rigidity, again, begins in Europe from five to six hours after death, and may continue from one to nine days. In Bengal the latest period of its commencement was seven hours, and the duration from four and a half hours to twenty-four hours. In Europe rigidity appears first in the muscles of the trunk and neck, in Bengal in the muscles of the neck and lower jaw. In both climates the muscles of the lower limbs are affected last. The order of disappearance of cadaveric rigidity appears much the same in both countries. A similar observation applies to cadaveric lividity. As regards the appearance of the discolouration of putrefaction, the time in Europe is from twenty-four to seventy-two hours. In Bengal the earliest period is seven hours and ten minutes, the average period twenty-six hours. In Bengal maggots may appears in twenty-four hours and eighteen minutes. In Europe vesications may present on the surface of the body in from fourteen to twenty days. In Bengal they may present in thirty-five hours. In Bengal gas may be formed and evolved in five hours and fifty minutes, while in Europe eight or ten days are required before gaseous products begin to be developed and to distend the abdomen. All these matters are important in Indian jurisprudence practice, and have never yet been thoroughly investigated. But climate causes great difference in such matters, and a thorough knowledge of this subject would materially aid Indian medical practitioners to a mature and accurate judgment in criminal cases in which the time of death has an important bearing on questions of guilt or innocence. At present Indian practitioners have to rely much on books and teaching of European authorities. Even the latest writer on Modern Medical Jurisprudence, Brigade Surgeon Lyon, C.I.E., has not very full information on the subject referred to above, and no comparison is made as regards differences arising from climate. Lyon states, as regards contractility, that there is no case on record where this stage has lasted 24 hours. But this remark does not evidently apply to Indian corpses especially. Similarly with reference to other changes. Brigade Surgeon Lyon's book is such an excellent one that we believe we have mentioned almost the only omission, and which we feel sure will be supplied in the next edition. It is too much the custom of the Indian Government to leave all kinds of medical investigation to the zeal of practitioners; but this matter, involving as it does questions bearing on guilt or innocence, should be taken up by Government, and investigated by a paid commission appointed under authority.

